# A colorimetric technique to characterize mass transfer during liquid-liquid slug flow in circular capillaries

**DOI:** 10.1016/j.mex.2021.101346

**Published:** 2021-04-22

**Authors:** Yanyan Liu, Chaoqun Yao, Lixia Yang, Mei Yang, Guangwen Chen

**Affiliations:** a Chinese Academy of Sciences, Dalian Institute of Chemical Physics, China; b University of Chinese Academy of Sciences, China

**Keywords:** Microreactor, Taylor flow, Liquid-liquid two phase, Dispersed droplets, Circular tubes

## Abstract

Continuous slug flow in microreactors are featured by alternative presence of regulate segments of immiscible phases in microchannel or capillaries with lateral dimensions below 1 mm. Due to the high interfacial area and short diffusive distance therein, such microreactors have been widely applied in chemical engineering processes that are sensitive to mass transfer. Therefore, mass transfer rates in microreactors have long been broadly investigated via either typical offline or online methods. Compared to these conventional methods, the colorimetric technique based on the oxidation of resazurin with oxygen enables direct determination of physical mass transfer rates. However, this technique was currently applied only to the gas-liquid system in microreactors, and mostly in rectangular channels due to the simplicity in image processing. Based on this, the current paper showed a demo where the colorimetric technique using resazurin was adapted to a liquid-liquid system for the mass transfer study of flowing droplets within a slug flow capillary. Experimental tips and tricks were summarized, and a sliced color-concentration calibration strategy was proposed to balance analyzing efficiency and accuracy.

## Graphical abstract

**Figure:**
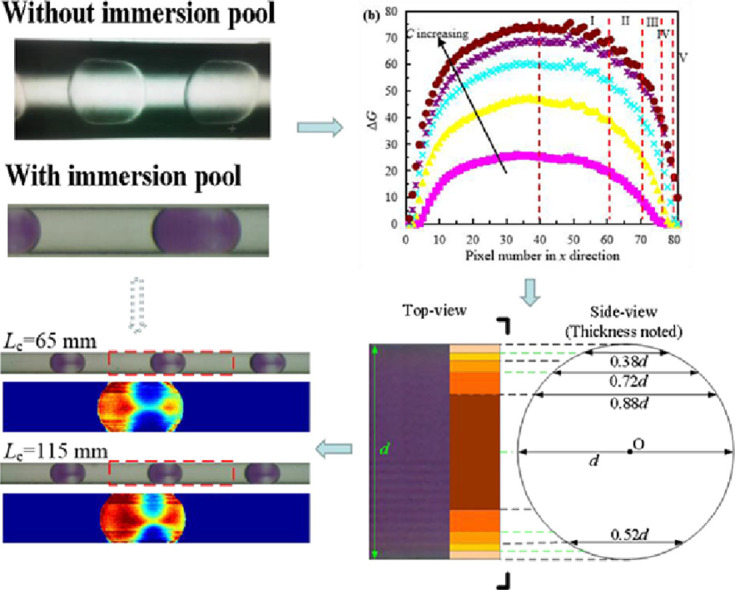

Specification table

**Table:** 

Subject Area:	Chemical Engineering
More specific subject area:	*Mass transport phenomena in microreactors*
Method name:	*Colorimetric technique of resazurin for mass transfer characterization*
Name and reference of original method:	*N. Dietrich, K. Loubière, M. Jimenez, G. Hébrard, C. Gourdon, A new direct technique for visualizing and measuring gas–liquid mass transfer around bubbles moving in a straight millimetric square channel, Chemical Engineering Science 100 (2013) 172–182.*
Resource availability:	*If applicable, include links to resources necessary to reproduce the method (e.g. data, software, hardware, reagent)*

## Method details

### Introduction

**Fig. 1:**
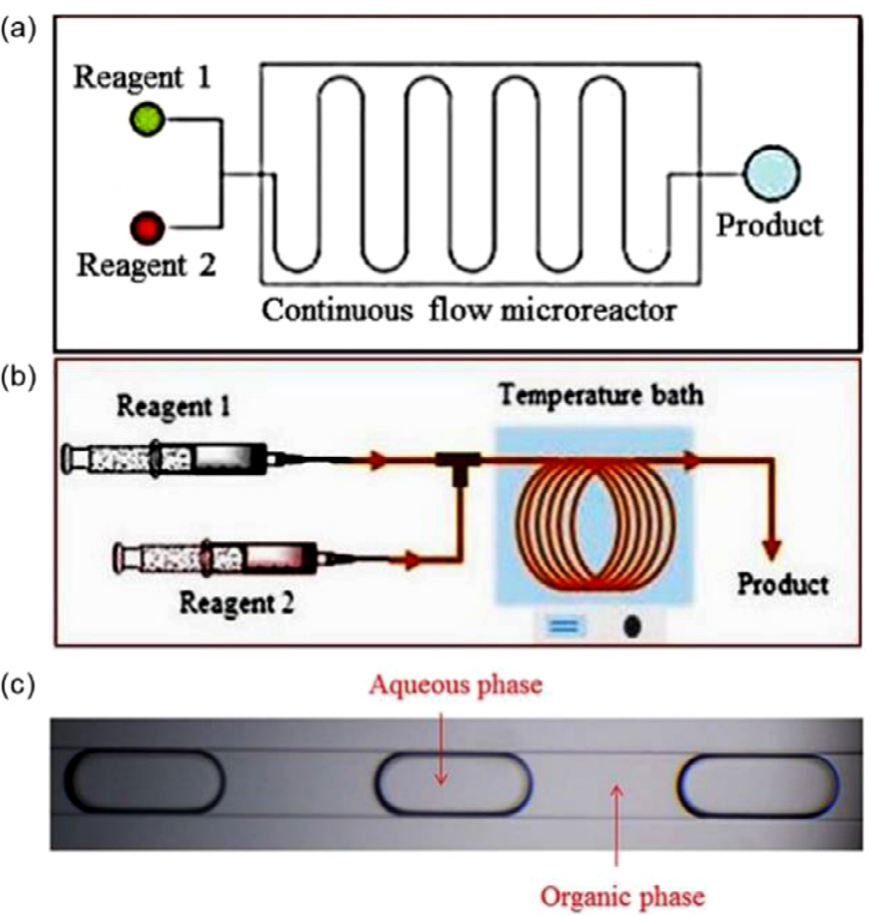
Microreactors taking the forms of in-chip microchannel (a) and capillary (b) [6], and (3) typical slug flow regime therein.

Continuous slug flow in microreactors are featured by immiscible phases flowing alternatively in the form of regulate segments in in-chip rectangular microchannels or circular capillaries (Fig. 1) with lateral dimensions below 1 mm. In such microreactors, a finer control over multiphase flow regime (i.e., lengths of involved segments) can be easily achieved by varying flow rate ratios [1]. Most importantly, due to the spatial confinement, the diffusion distance between phases is significantly shortened and larger interfacial area is created, resulting in mass transfer rates being 1–2 orders of magnitude higher than conventional reactors [2]. Thus, these microreactors have been widely employed in gas-liquid and liquid-liquid applications, such as fast reactions for higher selectivity and conversion [3,4] and particle preparations for narrower size distribution [5]. In these processes, the overall performances were highly sensitive to the mass transfer rate in both dispersed and continuous segments. Therefore, mass transfer in microreactors as one of the most important fundamentals is essential for process intensification.

Mass transfer characterization in microreactors can be achieved by either offline or online methods, which are originally adapted from conventional reactors. A most commonly used offline method is the physical absorption of CO_2_ to water or NaHCO_3_/Na_2_CO_3_ buffer solution to obtain the overall mass transfer coefficient *k*_L_*a* [2], where *k*_L_ and *a* respectively represent the mass transfer coefficient and the interfacial area. The interfacial area *a* can be determined either via the instantaneous reaction of CO_2_ with NaOH solution, or by calculating the bubble area according to recorded images [7,8]. An alternative is to alter the reaction rate constant between CO_2_ and CO_3_^2−^/HCO_3_^2−^ buffer solution by varying concentrations of the catalyst NaClO, resulting in a linear plot of the absorption rate against reaction rate constants, namely the Danckwerts’ plot. The slope and intercept of this plot respectively represent the physical mass transfer coefficient and the interfacial area. These offline methods are easy to carry out, but lack of accuracy especially in microreactors. The reason is the volumes of outlet tubes and/or sampling vials are usually comparable to that of the microchannel and/or capillaries, leading to prominent outlet effect towards mass transfer. Therefore, extra workload is usually involved to eliminate the outlet effect, such as parallel experiments in microreactors with and without main channel [2], sampling for various duration under a given flow rate [9] or both [10]. More importantly, local mass transfer information is missing, which largely limits the understanding of the mass transfer mechanism therein.

**Fig. 2:**
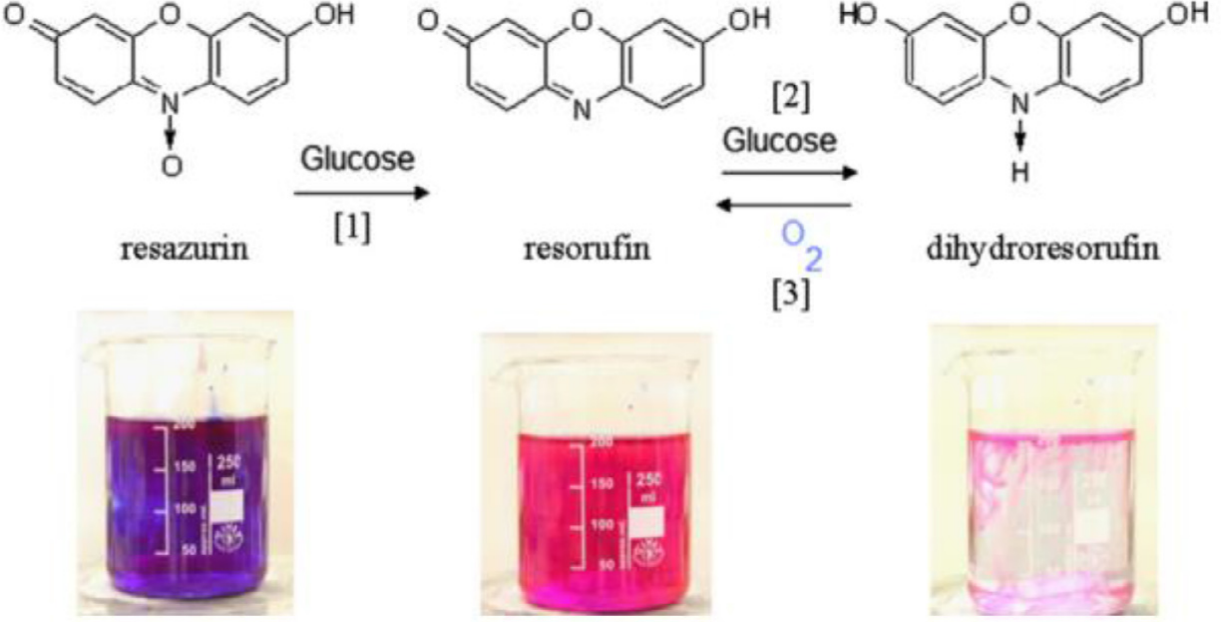
Oxidation-reduction reaction of resazurin in the aqueous solution [17]. (For interpretation of the references to color in this figure, the reader is referred to the web version of this article.)

Typical online methods include laser-induced fluorescence technique (LIF) and colorimetric technique, they both offer appealing advantages over those offline methods, such as high visual resolution, absence of end-effect and quick response time [11]. LIF technique involves the addition of a fluorescent dye, of which the fluorescence intensity under laser excitation decreases or increases with the concentration of certain compound (e.g., concentration of the dye itself or other ions like H^+^). According to the monotonic calibration curve between fluoresce intensity and the influencing compound, concentration field of the latter could be determined. The drawbacks of this technique include rather expensive equipment, complicated calibration caused by the inhomogeneity of laser light sheet, and confined observing area due to reflections and scattering of fluorescence at interface [12]. In comparison, colorimetric technique is more practical and easier to carry out, usually requiring just an LED backlight and a camera. Dessimoz et al. [13] and Yao et al. [14] employed pH indicators (bromothymol blue and phenol red) to determine the end-point of acid-base neutralization in liquid-liquid slug flow. Kockmann et al. [15,16] used the two-step oxidation of leuco-indigo carmine for local mass transfer and chemical selectivity study in gas-liquid reactions in capillaries. Note that results from these studies presented only the overall performance, which could not be decoupled from the effect of the reaction. In contrast, Dietrich et al. [17] developed a colorimetric method based on the oxidation reaction of resazurin with oxygen, which enables physical mass transfer study with simply optical microscope. As shown in Fig. 2, the purple resazurin solution can be gradually reduced by glucose and NaOH to pink resorufin (step 1) and finally to colorless dihydroresorufin (step 2). After being injected into the microchannel, dihydroresotufin was immediately oxidized to pink resorufin (step 3, O_2_+2·Dihydroresorufin→2·Resorufin + 2H_2_O) by oxygen molecules, leading to the color change of the aqueous solution, which was recorded by camera. By calibrating the relation between the amount of transferred oxygen and the color change (in grayscale) [15,17], the experimental amount of transferred oxygen was therein obtained. Most importantly, the large molecular size of resazurin makes it more difficult to diffuse in the liquid phase, resulting in a small enhancement factor (close to 1) [18]. This feature makes this resazurin system an easy and reliable option to investigate the physical mass transfer parameters within microreactors.

However, to the best of the authors’ knowledge, the colorimetric technique of resazurin system has only been used to investigate the mass transfer in gas-liquid slug flow. Whilst, liquid-liquid slug flow systems are even more commonly encountered in both research and industrial processes. Considering the wide usage of capillaries in the field of continuous flow processes (due to its low cost and high adaptability), here we presented a demo of the mass transfer study within dispersed droplets in a liquid-liquid slug flow capillary. Various tricks and precautions from experimental aspect were included, optical distortion and calibration problems caused by circular cross-section of capillaries were discussed, a sliced calibration strategy was used to improve analysis efficiency while maintaining the analyzing error around 10%.

## Overview of the colorimetric technique

**Fig. 3:**
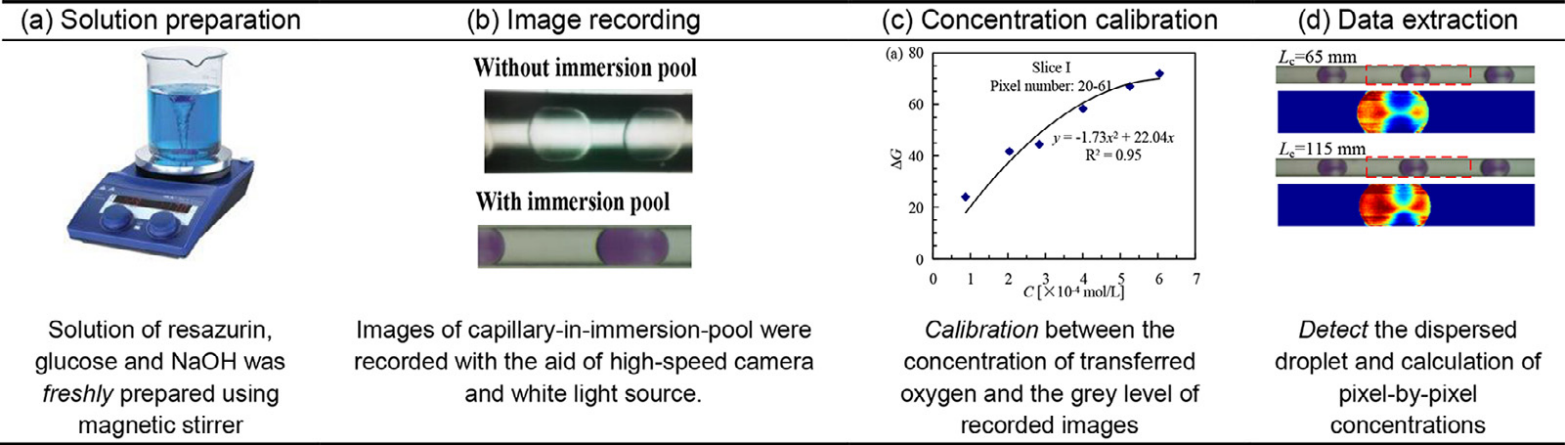
General procedures of colorimetric technique for mass transfer characterization.

Fig. 3 shows the general procedures of mass transfer characterization using the colorimetric technique, including (1) solution preparation, (2) image recording, (3) concentration calibration of the transferred oxygen through gray level of recorded images, and (4) data extraction. Among them, steps (2) and (3) are critical for the application specifically in capillary microreactors. Due to the differences of refraction indexes between the continuous liquid and air, images taken under ambient environment usually show black margins, i.e., optical distortion (Fig. 3b). To improve the clarity of images, an immersion pool was designed and fabricated to adjust the light intensity scattered near the capillary wall, details will be uncovered in the following text. Besides, the variation of tube thickness in radial direction caused various gray levels under given concentration of dihydroresorufin. Therefore, distinctive calibration curves should be plotted on behalf of experimental precision. Here, a five-sliced calibration strategy was introduced to improve processing efficiency and accuracy.

## Materials

### Chemicals

Resazurin (sodium salt, CAS 62758-13-8, molecular mass: 251.17 g/mol, purity ~80%) was purchased from Sigma Aldrich^Ⓡ^. Glucose (CAS 14431-43-7), sodium hydroxide (CAS 1310-73-2) and n-octane (CAS 111-65-9) were ordered from Aladdin^Ⓡ^. Deionized water was used to prepare the aqueous solution. N-octane was saturated with air before using.

### Equipment

• Two syringe pumps (LSP02-1B, LongerPump, China)
• Two glass syringes (50 mL, Ruize Fluid, JYQ-50-1, China)
• An optical microscope (SZX 16, Olympus, USA)
• A metal halide light source (MME-250, MORITEX SCHOTT)
• A high-speed CMOS camera (Phantom M310, Vision Research, USA, working at 100-500 frames/s)
• Two pieces of PMMA plates (length × width × depth = 140  × 60   × 10 mm)
• Tubing (PTFE, 0.5 mm ID, 1/16 OD; 1/16 ID, 1/8 OD)
• Magnetic Stirrers (IKA, C-MAG HS7)
• Pipet-Lite XLS manual single channel pipette (METTLER TOLEDO)
• Beaker (150 mL, Pyrex)
• Glass transparent reagent bottles with screw cap (250 mL, BFC)

## Pre-calculations

In order to obtain enough intensity of pink in recorded images, the concentration of reagent in aqueous solution were determined as 20 g/L glucose, 20 g/L NaOH and 0.3 g/L resazurin (i.e., *C*_resz,0_ = 1.20  ×  10^−3^ mol/L). The organic phase n-octane was saturated with air. For the described liquid-liquid slug flow, the location of mass transfer resistance and the enhancement factor of oxidation reaction were analyzed as followings:

### Location of mass transfer resistance

At room temperature (20 ± 2 °C) and ambient pressure (0.1 MPa), physical properties of both phases were listed in Table 1. The saturated concentration of oxygen in *n*-octane was *C*_O,0_ = 2.10  ×  10^−3^ mol/L [19], while its maximum consumption under full conversion of resazurin in the aqueous phase was *C*_max_ = *C*_resz,0_ /2 = 5.97 × 10^−4^  mol/L. That is to say, oxygen was in excess given comparable flow rates of two phases. The diffusivity of oxygen in organic phase *D*_O_ was reckoned by Stokes-Einstein equation, i.e., *D*_O_ = *D*_W_*μ*_W_/*μ*_O_ = 7.3  ×  10^−9^ m^2^/s [20], which was higher than that in the aqueous phase (*D*_W_ = 3.2  × 10^−9^ m^2^/s [21]). The partition coefficient of oxygen in the organic and aqueous phase was approximated by the ratio of their saturated concentrations in air, resulting in *m* = 10.08. Due to such high diffusivity and concentration in the organic phase, the mass transfer resistance in the liquid-liquid system lied in the aqueous phase.

**Table 1:** Physical properties of working fluids (20 ± 2 °C, 0.1 MPa).

Phase	Fluid	Viscosity*μ* [mPa·s]	Density*ρ* [kg/m^3^]	Surface tension*γ* [mN/m]
Organic	n-octane	0.565	702	49.67
Aqueous	0.3 g/L resazurin with 20 g/L glucose and 20 g/L sodium hydroxide	1.29	1002	-

### Estimation of enhancement factor

In order to obtain the physical mass transfer characterization with the colorimetric technique, the enhancement factor of the oxidation should be assured to be close to 1. According to Yang's doctoral thesis [22], the reaction constant for the oxidation of dihydroresorufin with oxygen molecules is *k*_2_=1.28 × 10^6^ L/(mol·s), then *Ha* number of oxidation of dihydroresorufin (step 3 in Fig. 1) under *C*_resz,0_ = 1.20  ×  10^−3^ mol/L could be calculated as

(1) $$Ha=\frac{\sqrt{k_{2}C_{resz,0}D_{W}}}{k_{L}}$$

Note that *k*_L_ theoretically equals to 1/(1/*k*_L,W_+1/*mk*_L,O_) for liquid-liquid system, where *k*_L,W_ and *k*_L,O_ are respectively the mass transfer coefficients from water and organic phases, but *k*_L_≈ *k*_L,W_ in current case, as explained above. For gas-liquid systems, *k*_L_ = *k*_L,W_ (mass transfer resistance in gas phase is usually negligible), and was reported to be in the magnitude of 10^−4^ m/s [11,21,23,24] in gas-liquid microreactors using this resazurin system. Thus, here we assume that the overall mass transfer coefficient *k*_L_ in our liquid-liquid system is in the same magnitude, leading to *Ha* = 17.7. Here, *Ha*>3 means that the oxidation is a fast reaction, i.e., the concentration of oxygen molecule at the interface is nearly 0.

Then the instantaneous enhancement factor could be further calculated by Eq. (2), where D_resz_ (= 8.65 × 10^−11^ m^2^/s [21]) is the diffusivity of dihydroresorufin in water, ν is the stoichiometric coefficient (= 2), and C* is the saturated concentration of oxygen in water (=2.55 × 10^−4^  mol/L [21]). Integrating Eqs. (2) to (3), the enhancement factor was obtained as *E* = 1.06, justifying that current system was suitable to quantify physical mass transfer coefficient.

(2) $$E_{i}=1+\frac{D_{resz}C_{resz,0}}{\nu D_{W}C^{*}}$$

(3) $$E=\frac{Ha\sqrt{\frac{E_{i}-E}{E_{i}-1}}}{\tanh\left(Ha\sqrt{\frac{E_{i}-E}{E_{i}-1}}\right)}$$

## Experimental setup

### Design and fabrication of immersion pool

**Fig. 4:**
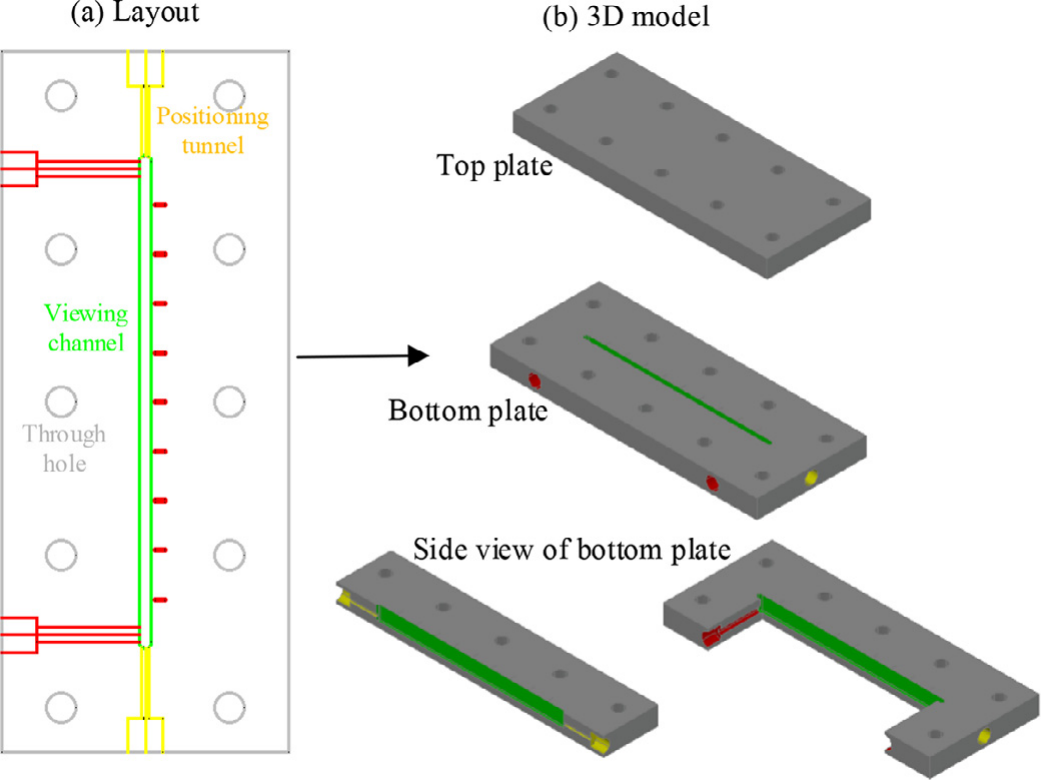
(a) Channel layout of the home-made immersion pool and (b) corresponding 3D models of the top and bottom plates. (For interpretation of the references to color in this figure, the reader is referred to the web version of this article.)

Fig. 4a shows the layout of the home-made immersion pool, which mainly includes three parts:

(I) The green part is a rectangular channel for flow viewing and image shooting (i.e., viewing channel), with dimensions being length × width × depth = 100  × 2.5  × 7 mm. The short red lines denote the length of the viewing channel in 1 cm interval.
(II) The yellow part is a circular tunnel composed of a wide and a thin section. The wide section contains screw threads at the inner wall to match the fitting and thus seals the port. The thin section serves as a positioning tunnel to straighten the capillary within the viewing channel, thus its diameter is slightly larger than the outer diameter of the applied capillary (i.e., 1.6 mm), being 1.8 mm.
(III) The red part is liquid delivery tunnel with similar structure to the yellow part, it was used to fill the immersion pool with the continuous liquid.

These structures described above were fabricated on a bottom PMMA plate, as shown in Fig. 4b. Then the bottom plate was joined with a top plate via 8 well-arranged through holes by nuts and bolts, in order to prevent liquid leakage. The design enables straightening the soft capillary tube and keeping different parts of the tube locating at the same depth. This is very important for the calibration, and is the prerequisite of the multi-layer sliced calibration, which will be discussed later.

### Setting up the recording system

**Fig. 5:**
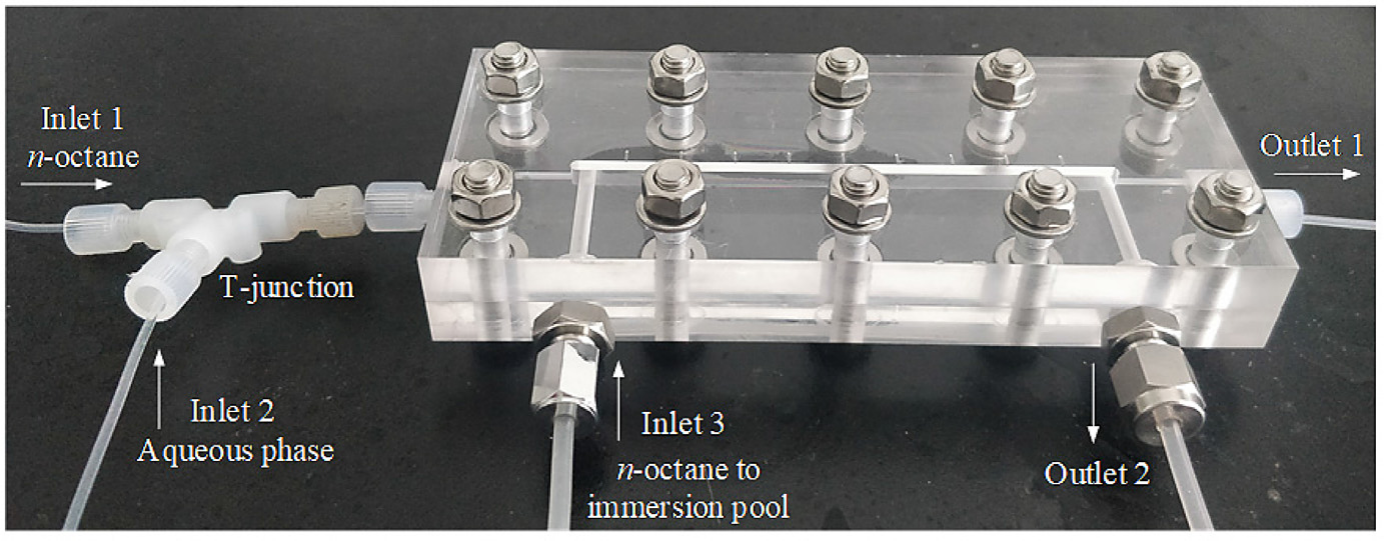
Image of assembled immersion pool and capillary microreactor, duplicated from [26].

**Note**: Aqueous solution of glycerol is a good alternative to the continuous phase used here, the exact concentration of glycerol could be determined by refractometer and observation over optical distortion of employed capillaries, refer to [25] for details.

Before conducting experiments, the capillary microreactor and shooting system should be set up as shown in Fig. 5. The recording system contains a light source, an optical microscope and a high-speed camera. Steps were summarized as follows:

(A) Insert the capillary tube through the immersion pool, straighten and tighten it at both ends of the described PMMA plate, with fittings and ferrules.
 **Note**: At least 5 cm long capillary should be left at one end of the PMMA plate, in order to connect to a Y/T-junction in the next step.
(B) Connect one end of the capillary to a T-junction which serves as inlets for fluids (here inlets 1 and 2 are respectively for organic and aqueous phases). Leave the other end of the capillary as outlet 1.
(C) Connect liquid delivery tunnel of the immersion pool to PTFE tubes (here with metal screws and nuts), as inlet 3 and outlet 2 shown in Fig. 5. Fill a syringe with the continuous phase (n-octane) and slowly inject it into the immersion pool through inlet 3, to expel gas bubbles therein.

(D) Lift the immersion pool up, letting the inlet 3 and outlet 2 face upward. Remove both screws and place a blind ferrule inside, then screws them up to prevent the continuous phase from leaking.
(E) Place the assembled PMMA plate and capillary between the back light and the high-speed camera (connected to the microscope).
(F) Set the magnification as × 2.0 (corresponding resolution 0.0071 mm/pixel) and adjust the camera till it focuses on the center-plane of the capillary. Thus, the view section of the capillary within the immersion pool spanned 81 × 1280 pixels.
 **Note**: Once everything is set up, keep it still without any further adjustment, till the end of the experiments. This is beneficial for the consistency of light intensity, as well as comparison and calculations among experimental images.

## Experimental protocol

(1) Prepare three solutions as listed below:

Solution A: 0.6 g/L resazurin solution (corresponds to 0.75 g/L resazurin product caused by impurities);
Solution B: 80 g/L glucose solution;
Solution C: 80 g/L NaOH solution.

**Note**: All solutions be contained in a sealed reagent bottle; solution B must be saved in fridge to restrain the activity of microorganisms.

(2) Place a 150 mL beaker containing a magnetic stir bar onto the magnetic stirrer, set the rotating rate to 500  r/min.

(3) According to the designated concentration of resazurin (Table 2), first add corresponding volumes of water, solution A and 20 mL solution B into the beaker with pipettes. Then, add 20 mL solution C drop by drop.

**Table 2:** Formulas of designated aqueous solutions used in current experiment.

Designated solution*	Designated concentration of resazurin (g/L)	Volume of deionized water (mL)	Volume of solution A (mL)	Volume of solution B (mL)	Volume of solution C (mL)
i	0 (Blank solution)	40	0	20	20
ii	0.040	34.7	5.3	20	20
iii	0.100	26.7	13.3	20	20
iv	0.200	13.3	26.7	20	20
v	0.260	5.3	34.6	20	20
vi	0.300	0	40.0	20	20

*Designated solution represents an aqueous solution with 20 g/L glucose, 20 g/L NaOH and a designated concentration of resazurin, the definite volume is 80 mL.

**Note**: Solution C must be added slowly, otherwise, side reactions may take place and cause color variation.

(4) Use the syringe to suck up a designated solution to around half of its range (~25 mL), let it rest till the solution was reduced into light green (takes about 15 min).

(5) For calibration, first block inlet 1. Next, fill the syringe with ambient air to its full range, shake and mix the air and liquid phase, then discharge the gas phase. Repeat the fill-shake-discharge cycle for at least three times, till the designated solution is completely oxidized to pink color. Then, immediately inject the solution into the setup and record corresponding images.

**Note**: Repeat steps 2–5 for each designated solution.

(6) For mass transfer characterization, unblock the inlet for organic phase. Suck the air-saturated n-octane with a glass syringe and connect it to the inlet.

(7) Prepare the designated solution (vi) with steps 2–3, and fill the syringe with the designated solution and connect it to the experimental setup as soon as possible. Let the liquid rest till solution is reduced to light green.

**Note**: The running time of the fully-reduced solution should be less than 1 h, in order to avoid the color change caused by side reactions. Thus, liquid volume in the syringe could be estimated by the flow rate and estimated running time.

(8) Inject both liquids into capillary microreactors with syringe pumps, and then record images with the setup described above.

## Results and discussion

### Calibration

**Fig. 6:**
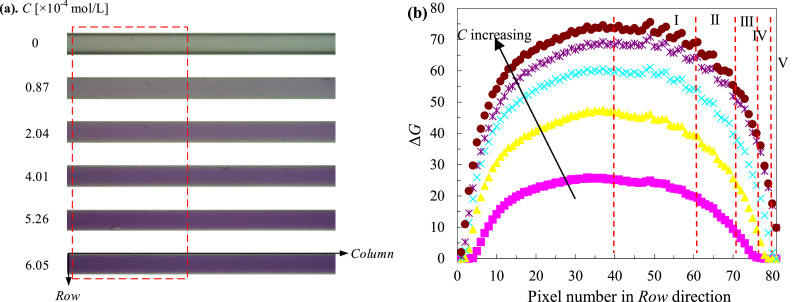
(a). Original images (row × column = 81 × 1280 pixels) for calibration under 6 designated solutions shown in Table 2, and (b) corresponding grey differences along row direction. The focused zone was shown in (a) with the red dotted box (81 × 500 pixels), duplicated from [26].

**Fig. 7:**
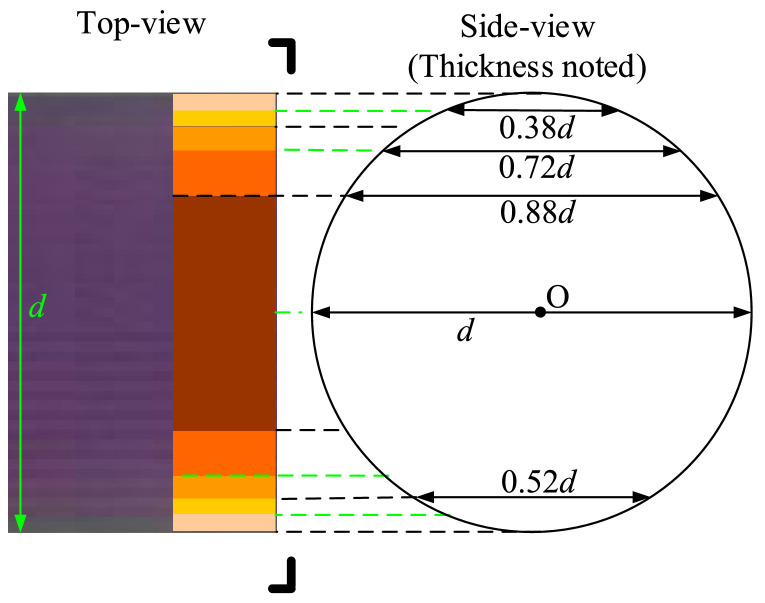
5-sliced calibration for capillary microreactor according to the thickness, which was denoted in the side-view, duplicated from [26].

To quantify the mass transfer performance, the molar concentration of transferred oxygen *C* (=0.5*C_resz_) was calibrated with the gray differences (Δ*G*) between corresponding images and the blank, leading to Δ*G*-*C* calibration curve(s). Typical images (81 row × 1280 column pixels) taken from the calibration experiments are shown in Fig. 6a, where 6 designated solutions were employed, including a blank solution (*C*_resz_=0 mol/L). In order to avoid the variation in light intensity along *column* direction, only 81 row × 500 column pixels (framed by the red box) were used for all calibrations and data extraction. For each designated solution, the gray difference in each row was averaged from at least 100 images, the variations of these Δ*G* values along the row number are shown in Fig. 6b. As can be seen, Δ*G* values under given solutions reached a plateau at the capillary center, and decreased dramatically near the channel wall. This is reasonable according to Lambert-Beer law, which states that the absorbance of light through a solution is related to both the concentration and optical path length. Fig. 7 illustrates that in current circular capillary, each row represents a small slice in original image, and corresponds to a certain optical length (or thickness) and cross-sectional area in side-view. Thus, multiple calibrations should be made in a row-specific fashion.

**Fig. 8:**
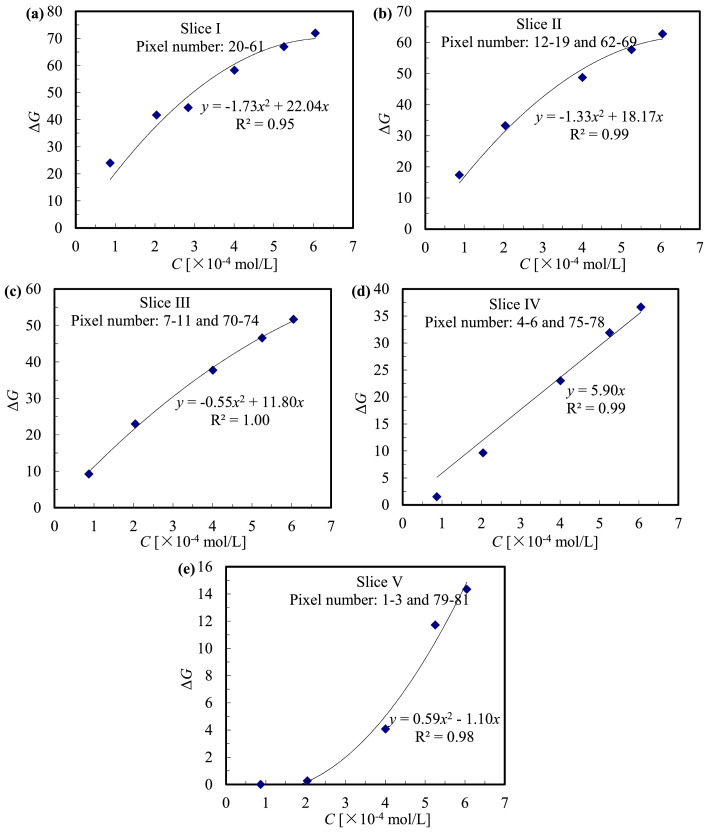
Calibration curves of five slices corresponding to Fig. 7, duplicated from [26].

**Note**: Current sliced-calibration strategy is only possible when the capillary has been well straightened. For curved capillaries, pixel-by-pixel calibration would be needed [24].

**Note**: Optimized number of slices may alter according to image resolution and required accuracy.

**Note**: Calibration curve must be monotonic in order to make quantitative calculation, but it may not be linear, depending on the superposition of mass transfer information within and outside of the focus planes [11] and dye concentration [27].

According to the variation rate of Δ*G* and corresponding thickness along the row number, the capillary area in each image was divided into 5 slices symmetrically, in order to improve the efficiency of data analysis (Figs. 6b and 7). Suppose the attenuation of light from the current system follows the linear approximation of Lambert-Beer law, then the error of calibration curves of the current 5-slice strategy equals to the deviation of thickness within each slice. Considering the differences in cross-sectional area of each slice *A*_slice_, the contributions of each slice to overall error could be estimated as: (Dev. in thickness) × (*A*_slice_*/A*_capillary_), which resulted in an estimated error of 13%, as summarized in Table 3. In fact, the calibration curves of five slices were either quadratic (thick slices, e.g., I and II) or linear (thin slices, e.g., IV), as shown in Fig. 8. Such 5-sliced calibration strategy is expected to further limit the estimated error. Because (1) the non-linear calibration curves in slices I-III were more accurate than the linear-assumption; (2) pixels near the capillary wall were significantly influenced by noises, compared to pixel-to-pixel calibration, current method would reduce such interference by involving and averaging more pixels; (3) actual error is also related to the distribution of transferred oxygen in the droplet. In current work, the transferred oxygen was mainly located around droplet caps, which means the error contribution at the droplet film (slice IV and V) was largely limited. Conclusively, the overall error is very likely to be lower than the estimated 13%.

**Table 3:** Estimated error of 5-sliced calibration method.

Slice	Row number	thickness	Deviation in depth	*A*_slice_/*A*_capillary_	overall error
I	20-61	1.0-0.88*d*	±6.4%	58.2%	3.7%
II	12-19, 62-69	0.88-0.72*d*	±10%	22.5%	2.3%
III	7-11, 70-74	0.72-0.52*d*	±16%	12.8%	2.0%
IV	4-6, 75-78	0.52-0.38*d*	±15%	4.1%	0.6%
V	1-3, 79-81	0.38-0*d*	±100%	2.4%	2.4%
				Total	13.0%

### Data extraction

**Fig. 9:**
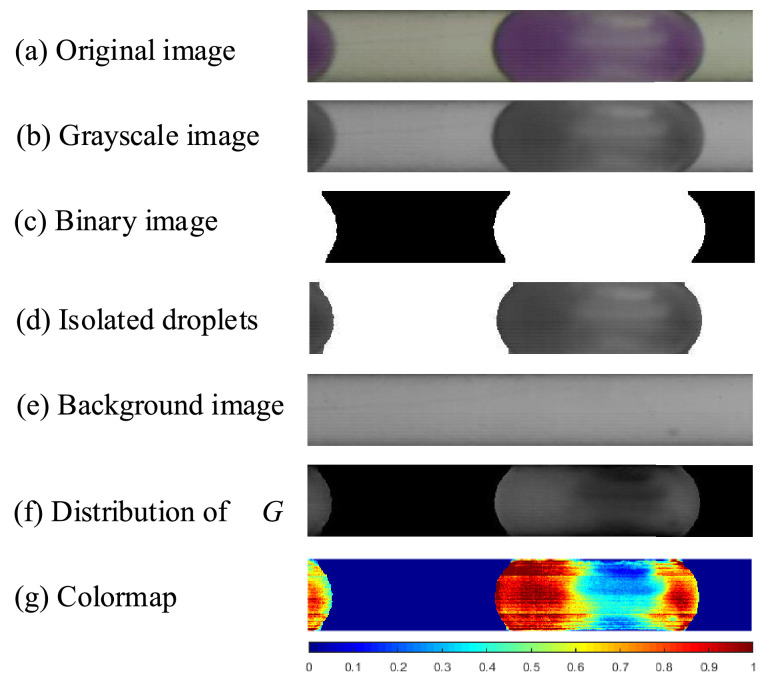
Image processing steps via *Matlab* for mass transfer characterization in flowing droplets.
  **Note**: The target area here should be consistent with the calibration area shown in Fig. 6a, to eliminate the possible errors from non-uniform distribution of light intensity; Conversion of resazurin in this area should be away from saturation.
  **Note**: When transferred oxygen is too little, only droplet caps could be detected. In this case, the whole droplet could be regrouped by matching and connecting adjacent droplet caps according to their radians.
(2) Isolate the grayscale droplets corresponding to these in the binary image (Fig. d).
(3) Convert the background image (with only blank solution flowing inside, Fig. 9e) into grayscale, and subtract Fig. 9c from it, obtaining the distribution of Δ*G* within droplet, while the Δ*G* values in the continuous slug section remain 0 (i.e., in color black in Fig. 9f).
(4) Convert Fig. 9f into a matrix, calculate the molar concentration of transferred oxygen *C* according to the row number of each pixel. Then normalize *C* with its maximum value *C*_max_ under complete oxidation, resulting in matrix of *C*_norm_ (= *C*/*C*_max_, in the range of 0–1).
(5) Generate a jet-mode colormap based on the above matrix, as shown in Fig. 9g.
  **Note**: the molar amount of transferred oxygen *n* within a droplet could be obtained by adding up the product of *C* and the volume of each pixel; dividing *n* by the droplet volume leads to the average concentration of transferred oxygen *C*_ave_ therein.
(6) The overall mass transfer coefficient could be derived by
  (4) $$k_{L}=\frac{C_{ave}J_{W}}{aL_{c}\Delta C_{m}}$$

According to the above *ΔG*-*C* calibration curves, the concentration of transferred oxygen in flowing droplets within a slug flow capillary could be extracted. As an example, the flow rates of aqueous and organic phases were both 0.6 mL/min. The data extraction processes in *Matlab* are list as follows:

(1) Crop the target area from original image, sequentially convert it to grayscale. By adjusting the threshold, further convert it to binary image in order to detect the droplets (Figs. 9a–c).

where *J*_W_ is the superficial velocity of the aqueous phase, *L*_c_ is the distance between inlet and center of focused area, *a* is the volumetric interfacial area, Δ*C*_m_ is the mean concentration difference between the inlet (indicated by subscript 0) and location *L*_c_ (subscript 1):

(5) $$\Delta C_{m}=\frac{\left(C_{O,0}/m-C_{W,0}\right)-\left(C_{O,1}/m-C_{W,1}\right)}{\ln\left(\frac{C_{O,0}/m-C_{W,0}}{C_{O,1}/m-C_{W,1}}\right)}=\frac{C_{O,0}-C_{O,1}}{m\ln\left(C_{O,0}/C_{O,1}\right)}$$

while *C*_O,1_ was obtained by mass balance within the focused area (i.e., *C*_O,0_*•Q*_O_ = *C*_O,1_*•Q*_O_ + *C*_ave_*•Q*_W_, *Q* represents volumetric flow rate).

When *J*_W_ and *J*_O_ were in the ranges of 0.034–0.068 m/s and 0.051–0.136 m/s, *k*_L_ was calculated to be 1.77 × 10^−4^ - 5.14 × 10^−4^  m/s [26]. These results are consistent with our initial assumption (in the magnitude of 10^−4^ m/s), meaning that these values could be seen as physical mass transfer coefficients. Please refer to Ref. [26] for further information such as mass transfer distribution.

## Declaration of Competing Interest

The authors declare that they have no known competing financial interests or personal relationships that could have appeared to influence the work reported in this paper.

The authors declare the following financial interests/personal relationships which may be considered as potential competing interests.
